# Secular trends in infant feeding practices during the first year of life in Norway: findings from 1998 to 2019 – the Spedkost surveys

**DOI:** 10.1017/S0007114523002246

**Published:** 2024-03-14

**Authors:** Anne Lene Kristiansen, Jannicke Borch Myhre, Mari Mohn Paulsen, Torunn Holm Totland, Britt Lande, Lene Frost Andersen

**Affiliations:** 1 Department of Nutrition, Institute of Basic Medical Sciences, University of Oslo, Oslo, Norway; 2 Department of Sports, Physical Education and Outdoor Studies, Faculty of Humanities, Sports and Educational Science, University of South-Eastern Norway, Bakkenteigen, Norway; 3 Department of Food Safety, Norwegian Institute of Public Health, Oslo, Norway; 4 Department of Physical Health and Ageing, Norwegian Institute of Public Health, Oslo, Norway; 5 Division for Prevention and Public Health, Norwegian Directorate of Health, Oslo, Norway

**Keywords:** Infant feeding practices, Breast-feeding, Solid foods, Norway

## Abstract

The aims of the present study were to assess secular trends in breast-feeding and to explore associations between age at introduction of solid foods and breast-feeding duration. Data from three national dietary surveys in Norway were used, including infants born in 1998 (Spedkost 1, *n* 1537), 2006 (Spedkost 2, *n* 1490) and 2018 (Spedkost 3, *n* 1831). In all surveys, around 80 % of the infants were breastfed at 6 months of age. At 12 months of age, breast-feeding rate was 41 % in Spedkost 1, increasing to 48 % in Spedkost 2 and 51 % in Spedkost 3. Compared with earlier introduction, introduction of solid foods at ≥ 5 months of age was associated with a lower risk of breast-feeding cessation during the first year of life in the two most recent Spedkost surveys. In Spedkost 2, the adjusted hazard ratio for breast-feeding cessation during the first year of life for those introduced to solid foods at ≥ 5 months of age was 0·43 (95 % CI (0·31, 0·60)), *P* < 0·001, while the corresponding number in Spedkost 3 was 0·44 (95 % CI (0·29, 0·67)), *P* < 0·001. In conclusion, breast-feeding at infant age 12 months increased over time. Introduction of solid foods at ≥ 5 months of age was positively associated with breast-feeding duration in the two most recent Spedkost surveys. As breast-feeding contributes to numerous health benefits for infant and mother, and possibly improved dietary sustainability in infancy, findings point to the importance of continued protection, support and promotion of breast-feeding.

In a life-cycle perspective, nutrition during the first 1000 d of life has been suggested to be of particular importance^(1–3)^. Breast milk is the ideal food for infants^(4)^, and breast-feeding contributes to numerous health benefits for infant and mother, both in the short term and in the long term^(5,6)^. Breast-feeding has a suggested low environmental footprint^(7,8)^ and by scaling up breast-feeding rates, dietary sustainability in early childhood is likely to improve. The economic benefit of breast-feeding is suggested to be of substantial value for societies^(9,10)^. The health, economic and environmental benefits of breast-feeding apply to populations in all countries and societies^(6,9,10)^, and the WHO highlights the importance of protecting, promoting and supporting breast-feeding^(11)^.

Since 2001, the WHO has recommended exclusive breast-feeding the first 6 months of life and thereafter a gradual introduction of appropriate complementary foods with continued breast-feeding for 2 years of age or beyond^(12,13)^. Most official guidelines also recommend exclusive breast-feeding the first 6 months of life^(14)^, including the Nordic countries^(15)^. Around infant aged 6 months, breast milk alone will no longer provide the nutritional needs for the infant, and complementary foods are required^(16)^. Most official guidelines commonly recommend that complementary foods should not be introduced before 4 months of age and no later than 6 months of age, and they are also generally consistent in supporting continued breast-feeding to at least 12 months of age^(14)^.

The Norwegian health authorities recommend exclusive breast-feeding for the first 6 months of life^(17)^. However, if food other than breast milk is needed, infant formula is the only alternative for the first 4 months. Solid foods may be introduced from 4 months of age if additional food to breast milk is needed. Furthermore, continued breast-feeding is recommended throughout the first year of life or longer^(17)^. The Norwegian Action Plan for a Healthier diet (2017–2021), extended to 2023, aims for a 50 % breast-feeding rate at 12 months of age^(18)^.

Even with the great advantages of breast-feeding, duration often falls short of recommendations^(19,20)^. Global time trend data, including data from 113 countries, show that any breast-feeding at 6 and 12 months of age has been stable from year 2000 to year 2019, with just below 90 % being breastfed at 6 months of age and around 81 % being breastfed at 12 months of age^(19)^. Data showed however large differences in rates according to country income, with the lowest breast-feeding rates observed in high-income countries. Vaz and collaborators included data from only high-income countries in the time period from 1986 to 2019^(20)^. Breast-feeding rates at 6 months of age, reported by twenty-six countries, varied from 4 % to 78 %, with a median rate of 45 %. Continued breast-feeding at 12 months of age was reported by twenty-five countries and rates varied from 0 % to 80 %, with a median rate of 29 %^(20)^.

Research has pointed to a wide range of determinants of breast-feeding^(9)^. The use of infant formula has long been associated with a decreased breast-feeding^(9)^, but less is known about other foods. Age at introduction of solid foods has been reported to influence length of breast-feeding^(21–24)^. However, exploring age at introduction of solid foods in relation to breast-feeding duration in the Norwegian setting has, to our knowledge, not previously been done.

Regular monitoring of infant feeding practices is important as it gives opportunities to assess status and changes in practices over time. Such insight can inform the knowledge base for health practitioners and policy makers on how to protect, support and promote favourable infant feeding practices. The aims of the present study were therefore to assess secular trends in breast-feeding and to explore associations between breast-feeding duration and age at introduction of solid foods. Data from three large national dietary surveys were used, conducted on infants born between 1998 and 2018.

## Methods

### Subjects and design

Secondary analyses with data from three large Norwegian population-based prospective cohort surveys, the national Spedkost surveys, were conducted. The surveys aimed to assess infant feeding practices and had a longitudinal design as they were carried out at infant aged 6 and 12 months. The first survey was conducted in 1998–1999 (Spedkost 1)^(25,26)^, the second in 2006–2007 (Spedkost 2)^(27,28)^ and the third in 2018–2019 (Spedkost 3)^(29,30)^. In all surveys, a nationally representative sample of about 3000 Norwegian infants was invited. All samples were drawn from the National Population Register.

In Spedkost 1 and Spedkost 2, the sample included all infants born in Norway during a 3-week period in April to May 1998 and 2006, respectively. In Spedkost 3, the sample included all infants born in Norway during a 4-week period in March 2018. It was assumed that the diet of infants born during these months was similar to the diet of infants born at other times of the year. More details about the samples have been described elsewhere^(25–30)^. For all samples, if the child was a twin or a triplet, the parents were asked only to include the oldest. Only infants born to mothers who themselves were born in Norway, Sweden or Denmark were included.

The Spedkost surveys were conducted according to the guidelines laid down in the Declaration of Helsinki, and all procedures involving human subjects were approved by the Regional Committees for Medical Research Ethics or the Norwegian Center for Research Data. Informed consent was obtained from the parent filling in the questionnaire. The Norwegian Center for Research Data approved the present study (ref. number 432 559). Those who declined to participate at infant aged 6 months were not invited to participate in the survey when the infant was 12 months of age.

The design of the Spedkost surveys has been published elsewhere^(25–30)^. Briefly, the mothers/parents received an invitation and a semi-quantitative FFQ or a link to the questionnaire by mail about 2 weeks before the child turned 6 and 12 months of age. In Spedkost 1, only paper-based questionnaires were available, while in Spedkost 2 and in Spedkost 3, the questionnaires were also available as web-based questionnaires.

In Spedkost 1 (at both 6 and 12 months of age) and Spedkost 2 (at 6 months of age), one combined thanks/reminder letter was sent to all participants. Additionally, one reminder letter with the questionnaire enclosed was sent to non-responders. Moreover, in Spedkost 2 (at 12 months of age) and Spedkost 3 (at both 6 and 12 months of age) non-responders were contacted by telephone and received one written reminder letter including the paper-based version of the questionnaire.

### Semi-quantitative FFQ

The questionnaires have been described in detail elsewhere^(25–30)^. In brief, they were designed to describe infant feeding practices at 6 and 12 months of age and to retrospectively describe feeding practices from birth up to the given age. In addition, the questionnaires included some questions about the characteristics of the infant and the parents.

The questionnaires used in Spedkost 1 were templates for the questionnaires used in Spedkost 2. Likewise, the questionnaires used in Spedkost 3 were based on the questionnaires used in Spedkost 2. All questionnaires were tested in pilot surveys and then revised. Additionally, the comparability of data obtained by the questionnaire used in Spedkost 1 and the questionnaire used in Spedkost 2 (among infants at 12 months of age) has been assessed and found acceptable^(31)^.

### Infant feeding practices and characteristics of infants and parents

For the present study, measurements of breast-feeding duration during the first year of life and age at introduction of solid foods were of main interest. Duration of breast-feeding was recorded in single weeks up to 7 weeks of age and in months from the age of 2 months up to 12 months of age. Breastfed infants included all infants who received breast milk, both those exclusively breastfed and those who had been introduced to other drinks than breast milk and/or solid foods. Breast milk intake was not quantified. Introduction to solid foods for the first time was recorded in single weeks up to 7 weeks of age and in half months from the age of 2 months up to 6 months of age. Solid food was defined as all other food except for water/infant formula/other milk/cordials/other drinks and dietary supplements. Solid food included gruel even if this was runny.

Data on infant and maternal characteristics were collected. Infant birth weight was reported as a continuous variable and categorised into < 2500, 2500–3500 and >3500 g. Gestational age was reported as a dichotomous variable: <38 weeks and ≥38 weeks. Maternal age, reported as a continuous variable, was categorised into ≤24, 25–34 and ≥35 years. Maternal marital status, reported by four categories, was recoded into married/cohabitant and not married/cohabitant. Four categories of maternal smoking at infant aged 6 months were recoded into ‘no’ and ‘yes’. Maternal education was reported by seven or eight categories and combined into low education (primary and secondary schools and tertiary vocational education) and higher education (college/university). Ten categories of maternal work situation at infant aged 12 months were combined into two categories: full-time employment and other (including part-time work, studies, mothers working at home/housewives, mothers on sick leave, unemployed, disabled, on rehabilitation, etc.). Four categories of parity were recoded into 1 child, 2 children and 3 children or more. Age at introduction of solid foods was categorised into ≤ 3·5 months of age, 4–4·5 months of age and ≥ 5 months of age. The most important reasons for introducing solid foods were only reported in Spedkost 3, at infant aged 6 months, with fifteen categories.

### Data analyses

All analyses were conducted using SPSS version 29 (IBM Corp. Released 2022. IBM SPSS Statistics for Windows, Version 29.0. IBM Corp.). Descriptive analyses and *χ* ^2^ test were used to explore secular trends in breast-feeding during the first year of life and to identify differences between the three Spedkost surveys in relation to the characteristics of the participants. Kaplan–Meier survival curves were used to examine the distribution of length of breast-feeding during the first year of life according to the age of introduction of solid foods. The log-rank was used to test differences between subgroups according to age at introduction of solid foods, and mean length of breast-feeding with 95 % CI was estimated. To evaluate the association between solid food introduction on the risk of subsequent breast-feeding cessation during the first year of life, Cox’s proportional hazard modelling, with adjustment for potential confounders, was conducted. As breast-feeding status is changing during the study period, only children who were still breastfed at the time of solid food introduction were included in analyses exploring age at introduction of solid foods and breast-feeding cessation. Infants fully fed with infant formula were not exposed to the study outcome and were therefore excluded. Immortal time bias is a consequence of either misclassification bias or selection bias^(32)^. A stratification on solid food introduced ≤ 3·5 months (no/yes), ≤ 4 months (no/yes) and ≤ 5 months (no/yes) was carried out in separate analysis to avoid immortal time bias. For each analysis, baseline was moved correspondingly.

## Results

For the present study, we included those who participated in the Spedkost surveys at both 6 and 12 months of age. Participants were 1537 parent/infant dyads (52 % response rate) from Spedkost 1, 1490 dyads (52 % response rate) from Spedkost 2 and 1831 dyads (64 % response rate) from Spedkost 3. In analyses exploring age at introduction of solid foods and breast-feeding cessation, only children who were still breastfed at the time when solid foods were introduced were included. Numbers in those analyses were 1156 parent/infant dyads from Spedkost 1, 1067 dyads from Spedkost 2 and 1464 dyads from Spedkost 3.

Table 1 presents the characteristics of the included participants. In all three surveys, there was roughly an equal number of boys and girls. At all three time points, most mothers were married/cohabitant. A larger proportion of the mothers was 35 years or older in Spedkost 2 and Spedkost 3 than in Spedkost 1. Maternal smoking at infant aged 6 months showed a large decline over time, from 28 % in Spedkost 1 to 4 % in Spedkost 3. Maternal education increased over time. In Spedkost 1, 48 % of the mothers had higher education, while this had increased to 66 % in Spedkost 2 and further to 74 % in Spedkost 3. Maternal full-time employment increased over time, from 18 % in Spedkost 1 to 28 % in Spedkost 2 and further to 47 % in Spedkost 3. Parity decreased over time, and a smaller proportion of the mothers had 3 children or more in Spedkost 3 than in Spedkost 1 and Spedkost 2.

**Table 1.** Characteristics of infants and their mothers participating in the present study (Numbers and percentages; mean values and standard deviation)

Characteristics	Spedkost 1 (*n* 1537)		Spedkost 2 (*n* 1490)		Spedkost 3 (*n* 1831)		*P* ^*^
	*n*	%	*n*	%	*n*	%	
Infant sex							
Boy	810	53	738	50	999	55	0·018
Girl	727	47	749	50	832	45	
	Mean	sd	Mean	sd	Mean	sd	
Infant birth weight, g	3634·2	581	3594·6	592	3544·0	557	0·272
	*n*	%	*n*	%	*n*	%	
<2500 g	52	3	56	4	76	4	
2500–3500 g	561	37	550	37	721	39	
>3500 g	914	60	880	59	1032	56	
Gestational age (weeks)							
<38	177	12	181	12	220	12	0·912
≥38	1326	88	1292	88	1607	88	
	Mean	sd	Mean	sd	Mean	sd	
Maternal age (years)^†^	29·5	4·9	31·2	4·8	31·2	4·6	**<**0·001
	*n*	%	*n*	%	*n*	%	
≤24 years	221	14	122	8	106	6	
25–34 years	1061	69	1003	68	1294	71	
≥35 years	253	17	346	24	431	24	
Maternal smoking^†^							
Yes	422	28	196	14	66	4	**<**0·001
No	1112	72	1256	86	1765	96	
Maternal marital status^‡^							
Married/cohabitant	1456	95	1409	96	1744	97	0·017
Not married/cohabitant	76	5	55	4	55	3	
Maternal education^‡^							
Lower education	784	52	494	34	461	26	**<**0·001
Higher education	723	48	970	66	1339	74	
Maternal employment^‡^							
Employed full-time	262	18	388	28	838	47	**<**0·001
Other	1190	82	1016	72	962	53	
Parity^‡^							
1 child	596	39	578	40	810	45	**<**0·001
2 children	557	37	594	40	715	40	
3 children or more	371	24	293	20	275	15	

* Chi-square test.

† Data reported at infant age 6 months.

‡ Data reported at infant age 12 months.

### Secular trends in breast-feeding

As presented in Table 2, breast-feeding rates were high across time. There were small differences between the three time points for breast-feeding rates at 6 months of age (*P* = 0·08). In Spedkost 1 and Spedkost 3, 77 % of the infants were breastfed at this age, while in Spedkost 2 80 % of the infants were breastfed at 6 months of age. Over time, a higher proportion of the infants were breastfed at 12 months of age (*P* < 0·001). In Spedkost 1, 41 % of the infants were breastfed at this age, while this proportion increased to 48 % in Spedkost 2 and further to 51 % in Spedkost 3.

**Table 2.** Data on breast-feeding and age at introduction of solid foods in the present study (Numbers and percentages)

Characteristics	Spedkost 1 (*n* 1537)		Spedkost 2 (*n* 1490)		Spedkost 3 (*n* 1831)		*P* ^*^
	*n*	%	*n*	%	*n*	%	
Breast-feeding at 6 months of age^†^							
Yes	1185	77	1187	80	1401	77	0·078
No	352	23	303	20	430	23	
Breast-feeding at 12 months of age^‡^							
Yes	634	41	701	48	938	51	**<**0·001
No	903	59	760	52	893	49	
Age at introduction of solid foods^†^							
≤ 3·5 months of age	333	24	155	12	104	6	**<**0·001
4–4·5 months of age	647	47	549	42	1129	63	
≥ 5 months of age	406	29	595	46	554	31	

* Chi-square test.

† Data reported at infant age 6 months.

‡ Data reported at infant age 12 months.

### Age at introduction of solid foods

In the period from 1998–1999 (Spedkost 1) to 2018–2019 (Spedkost 3), fewer infants were introduced to solid foods at ≤ 3·5 months of age (Table 2). In Spedkost 1, 24 % of the infants were introduced to solid foods at this age, while the corresponding numbers in Spedkost 2 and Spedkost 3 were 12 % and 6 %, respectively (Table 2). In Spedkost 1 and Spedkost 3, the most prevalent age for introduction of solid foods was at infant aged 4–4·5 months. In Spedkost 1, 47 % of the infants were introduced to solid foods at this age, while in Spedkost 3, the proportion was 63 %. In Spedkost 2, approximately the same percentage of infants had been introduced to solid foods by the age of 4–4·5 months (42 %) as those introduced to solid foods at the age of 5 months or older (46 %) (Table 2).

### Associations between age at introduction of solid foods and breast-feeding duration

**Fig. 1.**
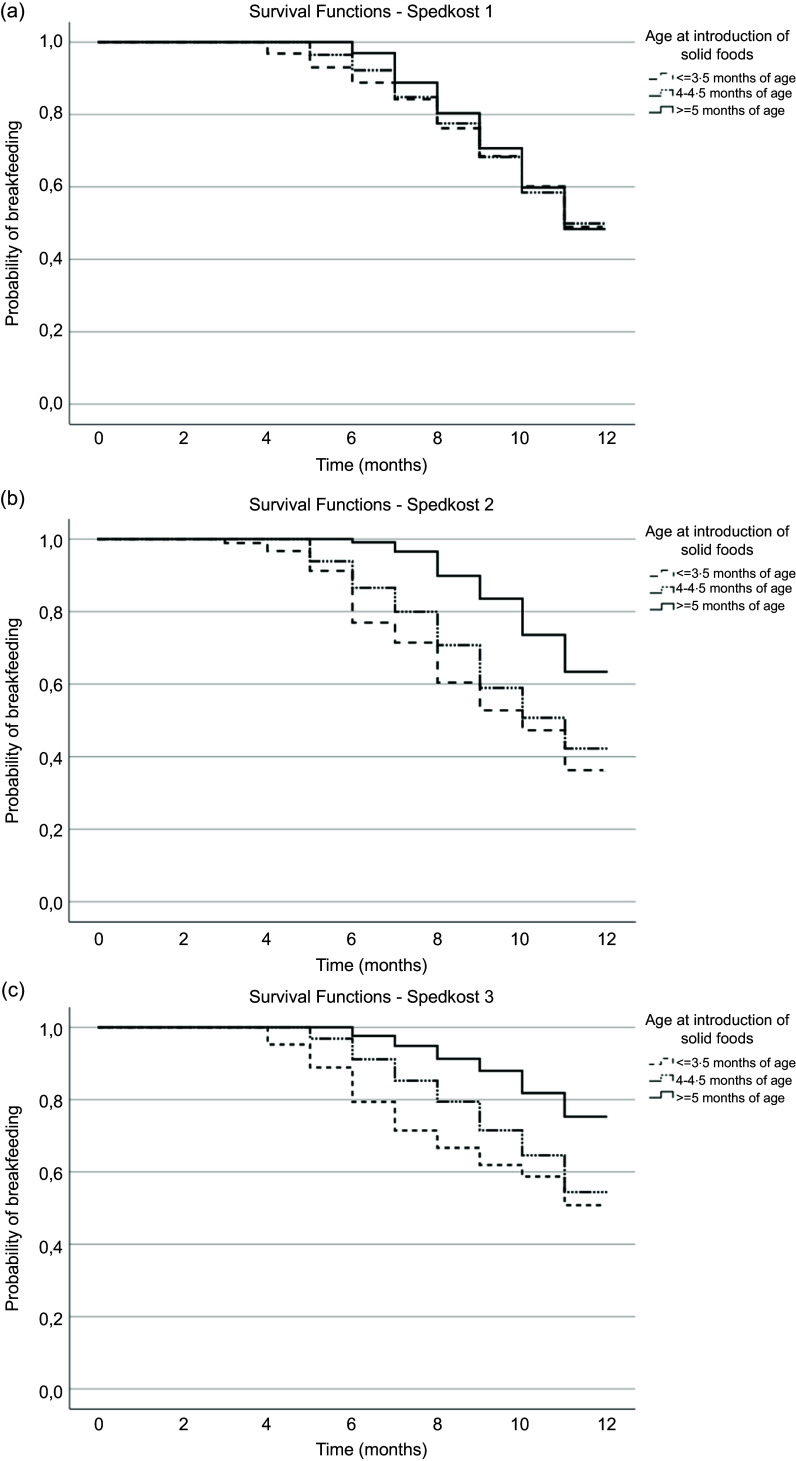
(a) Cumulative probability of being breastfed according to the age of introduction of solid foods in Spedkost 1 (*n* 1156). (b) Cumulative probability of being breastfed according to the age of introduction of solid foods in Spedkost 2 (*n* 1067). (c) Cumulative probability of being breastfed according to the age of introduction of solid foods in Spedkost 3 (*n* 1464).

Kaplan–Meier survival curves for breast-feeding duration according to age at introduction of solid foods are presented in Fig. 1(a–c). In Spedkost 1, the probability of being breastfeed during the first year of life according to age at introduction of solid foods did not differ between the three age groups (log-rank test *P* = 0·954, data not shown) (Fig. 1(a)). However, in Spedkost 2 and Spedkost 3, Kaplan–Meier survival curves revealed differences between groups (log-rank test *P* < 0·001 for both surveys, data not shown). Introduction of solid foods at ≥ 5 months of age compared with earlier introduction was associated with a larger probability of being breastfed during the first year of life (Fig. 1(b) and (c)).

In Spedkost 2, mean breast-feeding duration was 9·3 months (95 % CI (8·8, 9·9)) among those introduced to solid foods ≤ 3·5 months of age, while corresponding numbers were 9·8 months (95 % CI (9·6, 10·1)) and 11·1 months (95 % CI (10·9, 11·2)) among those introduced to solid foods at infant age 4–4·5 months and ≥ 5 months, respectively. In Spedkost 3, mean breast-feeding duration was 9·7 months (95 % CI (9·0, 10·4)) among those introduced to solid foods ≤ 3·5 months of age, while corresponding numbers were 10·4 months (95 % CI (10·3, 10·6)) and 11·3 months (95 % CI (11·2, 11·4)) among those introduced to solid foods at infant aged 4–4·5 months and ≥ 5 months, respectively.

Cox’s proportional hazard modelling, with adjustment for potential confounders, confirmed the results from the Kaplan–Meier analyses (Table 3). Compared with introduction of solid foods at ≤ 3·5 months of age, introduction of solid foods at ≥ 5 months of age was associated with a lower risk of breast-feeding cessation during the first year of life in the two most recent Spedkost surveys (*P* < 0·001). In Spedkost 2, the adjusted hazard ratio for breast-feeding cessation during the first year of life for those introduced to solid foods at ≥ 5 months of age was 0·43 (95 % CI (0·31, 0·60)), *P* < 0·001, while the corresponding number in Spedkost 3 was 0·44 (95 % CI (0·29, 0·67)), *P* < 0·001. In Spedkost 1, the adjusted hazard ratios were similar across groups (Table 3).

**Table 3.** Adjusted^*^ hazard ratio (HR) and 95 % CI for breast-feeding cessation during the first year of life (Hazard ratios and 95% confidence intervals)

Age at introduction of solid foods	Spedkost 1 (*n* 1156)			Spedkost 2 (*n* 1067)			Spedkost 3 (*n* 1464)		
	HR	95 % CI	*P*	HR	95 % CI	*P*	HR	95 % CI	*P*
≤ 3·5 months of age	1·00			1·00			1·00		
4–4·5 months of age	0·97	0·78, 1·21	0·785	0·88	0·64, 1·20	0·413	0·95	0·65, 1·41	0·812
≥ 5 months of age	0·98	0·77, 1·25	0·862	0·43	0·31, 0·60	**<**0·001	0·44	0·29, 0·67	**<**0·001

* Adjusted for these characteristics: infant sex, infant birth weight, gestational age, maternal age, maternal smoking, maternal marital status, maternal education, maternal employment and parity.

For stratification on various baseline time points for introduction of solid foods, results remained consistent; hence, immortal time bias was not considered to bias the results (data not shown).

## Discussion

The aims of the present study were to assess secular trends in breast-feeding and to explore associations between age at introduction of solid foods and breast-feeding duration. Trends in breast-feeding from 1998–1999 to 2018–2019 indicated that rates at 6 months remained stably high across time, while there was an increase in breast-feeding rates at 12 months. Compared with introduction of solid foods at ≤ 3·5 months of age, introduction of solid foods at ≥ 5 months of age was associated with a lower risk of breast-feeding cessation during the first year of life in the two most recent Spedkost surveys.

### Secular trends in breast-feeding

Breast-feeding rates at 6 months of age remained stably high (77–80 %) over time in this 20-year period, while breast-feeding rates at infant age 12 months increased from 41 % to 51 %. Prevalence of breast-feeding in the Nordic countries has traditionally been high. In Finland, increasing breast-feeding rates have been observed from 1995 until 2019^(33)^. Breast-feeding at 6–8 months of age increased from 40 % in 1995 to 76 % in 2019, while breast-feeding at infant age 9–12 months increased from 25 % to 62 % in the same period^(33)^. In Sweden, breast-feeding rates were at the highest in the period from 1995 to 2004^(34)^. The breast-feeding rate at 6 months of age was around 72 % in this period, while from 2010 to 2019 it was around 63 %. Still, breast-feeding rates at 12 months of age increased from 16 % in 2010 to 28 % in 2019^(34)^.

One important structural determinant for breast-feeding is maternal leave policy^(9)^. In Norway, the parental leave period increased in the period when the Spedkost surveys were conducted and could contribute to explain the increase in breast-feeding rates observed at infant aged 12 months. In 1998–1999 when the Spedkost 1 survey was conducted, the 100 % parental leave period was 42 weeks, while this had increased to 44 weeks in 2006–2007 and further to 49 weeks in 2018–2019 when the Spedkost 3 survey was conducted^(35)^.

### Age at introduction of solid foods and reasons for introduction of solid foods

Over time, fewer infants were introduced to solid foods at ≤3·5 months of age. When the first Spedkost survey was conducted in 1998–1999, Norwegian health authorities recommended a gradual introduction of solid foods at 4–6 months of age^(36)^. In 2001, the infant feeding recommendations were revised and introduction of solid foods was recommended from 6 months of age^(37)^. Revised recommendations were also launched in 2016^(17)^. The recommendation was still introduction of solid foods at 6 months of age, but it was made clearer that solid foods can be introduced from 4 months of age if needed. However, at all three time points, it was recommended that solid foods should not be introduced before 4 months of age. It seems as if the differences in recommendations were reflected in age at introduction of solid foods found in the present study. Lessa and co-workers also observed changes in age at introduction of solid foods across time^(21)^. In the earliest study, including infants born in 1991–1992, 68 % of the infants had been introduced to solid foods before 4 months of age, while in the most recent study, including infants born in 2010–2011, 21 % had been introduced to solid foods at this age^(21)^. From 2003, the UK. Department of Health recommended introduction of solid food from 6 months of age, from the previous recommendation of 4–6 months of age^(21)^.

In the Spedkost 3 survey, more than 60 % of the infants were introduced to solid foods at 4–4·5 months of age. The top three reasons for introducing solid foods, reported at infant age 6 months in the Spedkost 3 survey, were that the infant showed great interest in food; the parents thought the infant was old enough and that they wanted the infant to be accustomed to new consistencies and flavours (data not shown). This is in line with the reviews by Harrison *et al*.^(38)^ and by Spyreli *et al*.^(39)^. Most of the data included in the reviews were from UK, USA and Australia, and they found that ‘baby-driven cues’ were typical reasons for solid food introduction^(38,39)^. The reviews reported a wide range of factors associated with the introduction of solid foods to infants^(38,39)^, and knowledge of such factors can be important to inform the development within infant feeding promotion efforts and contribute to identify the needs within the field and thereby possibly improve compliance with infant feeding recommendations.

### Associations between age at introduction of solid foods and breast-feeding cessation

Compared with introduction of solid foods at ≤ 3·5 months of age, introduction of solid foods at ≥ 5 months of age was associated with a lower risk of breast-feeding cessation during the first year of life in the two most recent Spedkost surveys. Age at introduction of solid foods has previously been reported to influence length of breast-feeding. A study among low-income women in Canada reported that late introduction of solid foods was the strongest predictor of the duration of breast-feeding^(23)^. In UK, Lessa and collaborators^(21)^ found that the risk of terminating breast-feeding before 6 months of age was highest among mothers introducing solid foods before infant aged 4 months and lowest among mothers introducing solid foods at infant aged 5 months and older. This was found in all three cohorts, including infants born in the period from 1991 until 2011^(21)^. An Australian study from 2002 to 2003 observed that those introduced to solid foods at or after 17 weeks of age were breastfed for an average of 11 weeks longer compared with those introduced to solid foods before 17 weeks of age^(22)^. A recent study from Sweden, including 1251 participants in the period from 2012 to 2015, reported that even tiny tastings of solid foods (1 ml) were associated with a shorter duration of breast-feeding during the first year of life^(24)^. However, another Swedish study including highly motivated mothers with previous breast-feeding experience, having infants born in the period from 1989 to 1992, found no significant associations between infant age at introduction of solid foods and breast-feeding duration^(40)^. It could be that mothers facing challenges with breast-feeding are the ones introducing solid foods the first. A recent Norwegian study among 715 mothers, with infants born in 2015–2016, reported that ‘infants who were given a combination of breastmilk and formula at three months of age had lower odds of later introduction to solid food compared to infants who were given breastmilk as milk type at three months of age’^(41)^.

### Strengths and limitations

The present study comes with some limitations. The data represent a cross-sectional nature and a limitation of this is that no causal relationship can be drawn from this study. During the last 20 years, the population in Norway has changed^(42,43)^, and this was also reflected in the present study. As in the general population, maternal educational level increased, mothers were older, mothers gave birth to fewer children and fewer were smokers across time. Additionally, those participating in the Spedkost surveys tend to have a higher educational level compared with the general population in Norway at the time when the surveys were conducted^(25–30)^. It might be that our sample deviates from a representative sample and shows higher breast-feeding rates than what is the actual situation. At last, as the Spedkost surveys do not include infants born by mothers outside of Scandinavia, this might have led to missing out some real changes across time. A strength of our study is the large sample size included at each time point, that the studies were national dietary surveys and that three surveys were included. Data were also collected close to the time for breast-feeding cessation and age at introduction of solid foods, which might have reduced the risk of recall bias. Additionally, across time the questionnaires were collected at the same infant age, survey techniques were similar and the infant feeding variables were highly comparable.

In conclusion, breast-feeding at infant aged 12 months increased over time. Introduction of solid foods at ≥ 5 months of age was positively associated with breast-feeding duration in the two most recent Spedkost surveys. As breast-feeding contributes to numerous health benefits for infant and mother, and possibly improved dietary sustainability in infancy, findings point to the importance of continued protection, support and promotion of breast-feeding.
